# Partial appendiceal duplication with double bases converging into a single body and tip: a first reported case from Nepal

**DOI:** 10.1097/MS9.0000000000005396

**Published:** 2026-07-21

**Authors:** Raj Kumar Rajbanshi, Sanish Pokhrel, Chandan Lagarwar, Soni Roy, Rahul Yadav, Krishna Thapa

**Affiliations:** a Department of Surgery, Universal Colleges of Medical Sciences and Teaching Hospital, Siddharthanagar, Nepal; b Department of Emergency, Universal Colleges of Medical Sciences and Teaching Hospital, Siddharthanagar, Nepal

**Keywords:** appendectomy, case report, inverted Y-shaped appendix, partial appendiceal duplication

## Abstract

**Introduction:**

Anomalies of the appendix are very rare, occurring only in 0.004% to 0.009% of appendectomy cases. Among these, appendiceal duplication represents a particularly unusual finding. We are presenting a case of an appendicular lump with an appendix having two bases converging into one body and tip.

**Case presentation:**

A 50 year-old female presented to a tertiary care center in Nepal with chief complaints of abdominal pain for 3 days and vomiting for 3 days. The abdominal pain was acute in onset, sharp, and aching, located in the right iliac region. Ultrasonography revealed that the appendix was dilated at the region of the tip and body (approximately 9–10 mm) with a normal-appearing base. The patient was planned for an interval open appendectomy for an appendicular lump. Intraoperatively, the appendix was found to have two luminal bases with a single tip and body.

**Discussion:**

Partial duplication of the appendix with an inverted Y-shaped configuration is an exceptionally rare anatomical anomaly. Such duplications are mostly discovered incidentally during surgery. Their clinical significance lies in their potential to cause diagnostic confusion and incomplete management if unrecognized.

**Conclusion:**

This case highlights the importance of careful intraoperative examination of the cecal region during appendectomy procedures to avoid missed diagnoses, repeated surgeries, and potential medicolegal implications.

## Introduction

Anomalies of the appendix are very rare, occurring in only 0.004–0.009% of appendectomy cases^[^1^]^. Among these, appendiceal duplication represents a particularly unusual finding. Several classification systems have been proposed, most notably the modified Cave–Wallbridge classification, which categorizes appendiceal duplication into Types A–D based on anatomical configuration and cecal association^[^2^]^. We are presenting a case of an appendicular lump with an appendix having two bases converging into a single body and tip. To our knowledge, this precise anatomical pattern has not been explicitly described in existing classification systems. This is the first case reported from Nepal. Reporting such cases is important to enhance surgical awareness, prevent missed diagnoses, and reduce the risk of repeat surgery or medicolegal consequences. This case report has been prepared in line with the SCARE Checklist 2025 and TITAN Guidelines 2025^[^3,4^]^.

HIGHLIGHTS

- Anomalies of the appendix are very rare, occurring in only 0.004–0.009% of appendectomy cases. Among these, appendiceal duplication represents a particularly unusual finding.
- The appendix had two lumen bases with a single tip and body.
- This is the first case reported from Nepal with two lumen bases and a single body and tip of the appendix.

## Case presentation

**Figure 1.**
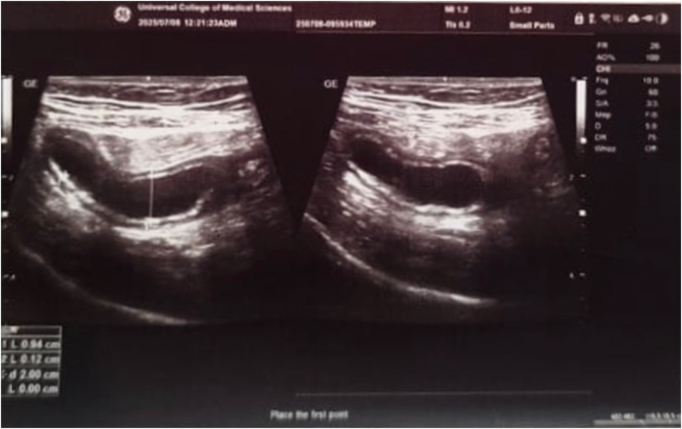
The appendix is dilated at the region of the tip and body (approximately 9–10 mm) with a normal appearing base. The patient was managed conservatively initially and was prepared for an interval open appendectomy for an appendicular lump.

A 50 year-old female presented to a tertiary care center in Nepal with chief complaints of abdominal pain for 3 days and vomiting for 3 days. The abdominal pain was acute in onset, sharp, and aching, located in the right iliac region, non-radiating, with no aggravating or relieving factors. Vomiting was present, with two episodes containing food particles, non-bilious and non-blood-stained, with no aggravating factors and relieved after taking antiemetic drugs. She had normal bladder and bowel habits. There was no history of fever, burning micturition, or vaginal discharge. The patient had a past history of pulmonary tuberculosis, for which she underwent anti-tubercular therapy for 6 months. She had undergone bilateral tubal ligation for family planning. There was no history of hypertension, diabetes, chronic obstructive pulmonary disease, thyroid disorders, heart diseases, or any other genetic disorders. All vital signs were normal. On examination, there was tenderness in the right iliac fossa with muscle guarding. Investigations were conducted, with CBC showing hemoglobin at 12.2 mg/dl, WBC at 5400 cells/cumm, and platelets at 307 000 cells/cumm. The differential count showed neutrophils at 46%, lymphocytes at 7%, eosinophils at 1%, monocytes at 6%, and basophils at 0%. The renal function test (RFT) was within the normal range. The liver function test (LFT) showed total bilirubin at 1.6 mg/dl, direct bilirubin at 0.6 mg/dl, indirect bilirubin at 1 mg/dl, AST at 83 IU/L, ALT at 92 IU/L, ALP at 49 IU/L, total protein at 8 gm/dl, and serum albumin at 5.2 gm/dl. Ultrasonography revealed that the appendix was dilated at the region of the tip and body (approximately 9–10 mm) with a normal-appearing base. The wall was normal in thickness, with no surrounding omental echogenicity, collection, or periappendiceal collection (Fig. 1).

Based on the patient’s right iliac fossa pain with localized tenderness and guarding, the primary differential diagnoses included acute appendicitis with an appendicular lump, ileocecal tuberculosis (given the past TB history), cecal diverticulitis, and gynecological pathology. A normal leukocyte count and the absence of systemic toxicity favored a localized inflammatory process rather than perforation or generalized peritonitis. Ultrasonography demonstrating a dilated appendiceal body and tip with a normal base supported the diagnosis of appendicitis with an appendicular lump, leading to the decision for interval appendectomy.

In the operation theater, under all aseptic conditions, a Lanz incision was made, and the appendix was localized. The appendix had two lumen bases with a single tip and body. The mesoappendix was ligated and separated from both bases; both appendix bases were tied, and dissection was performed. Hemostasis was secured, and the abdominal wall was closed in layers. The appendix was sent for histopathological examination.

## Histopathological reports

### Gross findings

The specimen, received in formalin, consisted of a single tubular structure, light brown in color and firm in consistency, measuring 5 × 1 cm. The specimen demonstrated an identifiable tip, body, and base, with two distinct luminal openings at the base. On the cut section, the lumina were patent and not occluded by a fecalith. The second luminal opening at the base measured approximately 0.7 × 0.5 cm.

### Microscopic findings

Sections submitted show mucosa lined by tall columnar cells with basally placed nuclei, admixed with goblet cells. The underlying lamina propria is fibrocollagenous and consists of lymphoid follicles, mucosal glands, and mixed inflammatory infiltrates comprising neutrophils, lymphocytes, and eosinophils. The submucosa shows lipomatization. The muscular layer is infiltrated by mixed inflammatory cell infiltrates comprising neutrophils, lymphocytes, and eosinophils. The subserosa and serosa consist of congested blood vessels, hemorrhages, and mature adipocytes. No atypia or granulomas are seen in the sections studied.

Histopathological images could not be retrieved; however, comprehensive gross and microscopic histopathological examination confirmed the diagnosis.

This is the intraoperative view of the appendix.

The patient was stable, shifted to the ward, and discharged after the wound healed. The patient was followed up 30 days post-operatively. She remained clinically stable with complete wound healing. There were no post-operative complications. The patient had resumed normal daily activities and did not require further intervention.

## Discussion

**Figure 2.**
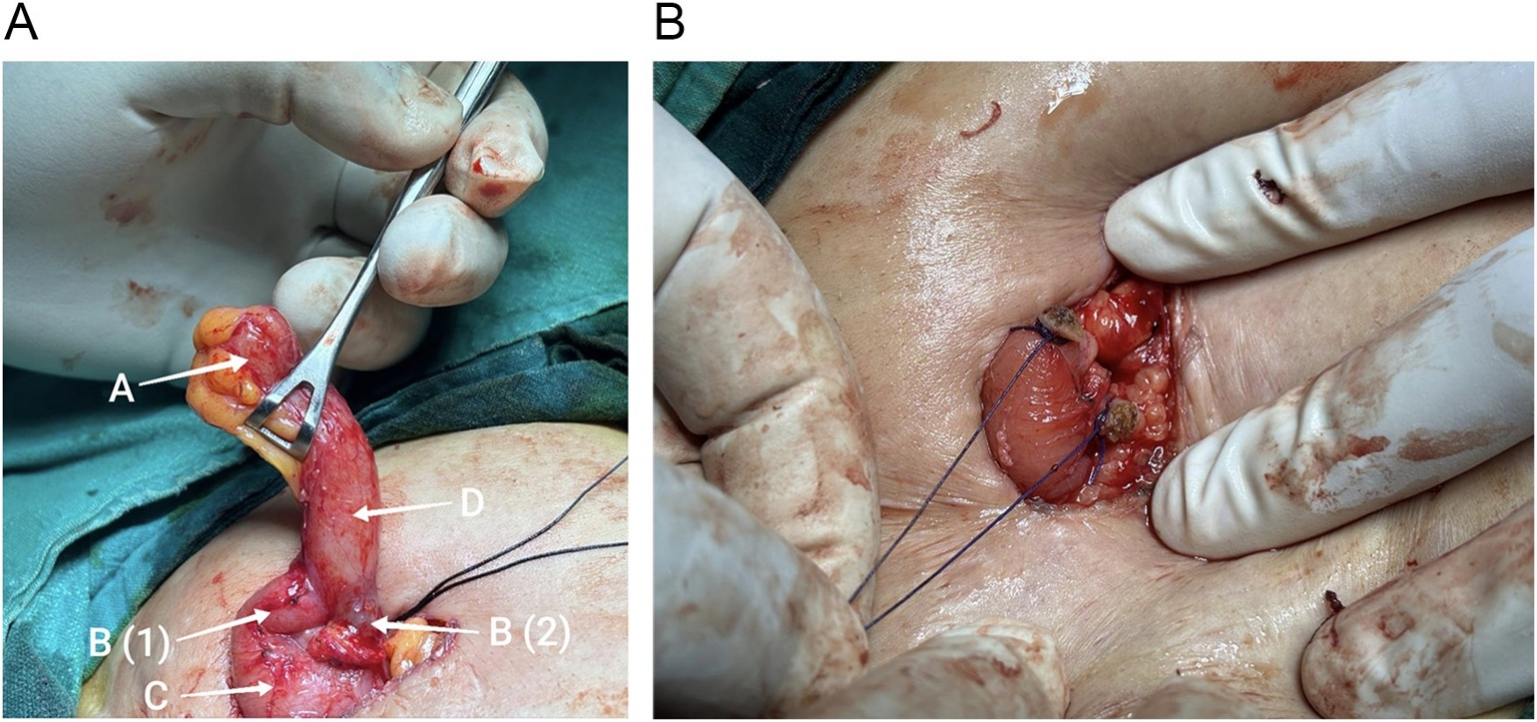
(A) showing: A - Tip; B(1) - First base; B(2) - Second base; C - Cecum; D - Body. (B) showing post-appendectomy stump of the two bases of the appendix.

The appendix develops as an outpouching of the cecum by the 8th week of gestation, following gut rotation that places it in the right lower quadrant. While its normal embryology is understood, the cause of duplication remains unclear. Literature suggests two possible mechanisms: persistence of a transient embryonic structure or a broader midgut abnormality. Although these theories explain some types of duplication, they are insufficient to explain all types of duplication that exist^[^5^].^ The vermiform appendix can exhibit anatomical variations and congenital abnormalities that can complicate the diagnosis of acute appendicitis. Although rare, anomalies like appendix agenesis or duplication can sometimes lead to diagnostic and management confusion^[^6^]^. Appendiceal duplication can occur alone or with cecal duplication and is classified by the modified Cave-Wallbridge system into four types, Types A to D. Type A involves partial duplication of a single appendix. Type B includes a normal appendix plus a second one in various locations (B1: opposite the ileocecal valve, B2: along the tenia coli, B3: hepatic flexure, B4: splenic flexure). Type C features cecal duplication with one appendix on each. Type D, the rarest, is a horseshoe-shaped appendix with two openings into the cecum^[^2^]^. Our case has an inverted Y shape, partial duplication with two separate bases merging into a single body and tip, an anatomical variation that has not been clearly classified in existing classification systems to date (Fig. 2A and B).

Among 141 cases of appendiceal duplication that have been reported, most are diagnosed intra-operatively, with a male-to-female ratio of 1.4:1. While most GI duplications present before the age of 2 years, duplicated appendix cases range from fetal life to 69 years of age, with type A being the most common^[^7^]^.

Surgeons should routinely inspect the cecum during appendectomy to rule out appendiceal duplication, as missing it can lead to repeat surgery, complications, and medicolegal issues^[^8^]^. Though rare, duplicated appendix should be considered in patients with lower abdominal pain, even in post-appendectomy status. Differential diagnoses include various gastrointestinal and genitourinary conditions. Differential diagnosis includes a congenital cecal diverticulum, Meckel’s diverticulum, gastroenteritis, acute mesenteric adenitis, intussusception, inflammatory bowel disease, epiploic appendagitis, stump appendicitis, colonic adenocarcinoma, and genitourinary pathology^[^9^]^. Duplication of the appendix is often asymptomatic but can present as acute appendicitis. Rarely, it can mimic colon cancer or cause small bowel obstruction. Investigations like barium studies can detect it but are not done routinely, so most cases are found incidentally during surgery, autopsy, or histology. It’s important not to confuse appendiceal duplication with cecal or appendiceal diverticulum, which lack lymphoid tissue on histology^[^10^]^.

## Conclusion

Partial duplication of the appendix with an inverted Y-shaped configuration is an exceptionally rare anatomical anomaly. Such duplications are mostly discovered incidentally during surgery. Their clinical significance lies in their potential to cause diagnostic confusion and incomplete management if unrecognized. This case highlights the importance of careful intraoperative examination of the cecal region during appendectomy procedures to avoid missed diagnoses, repeat surgeries, and potential medicolegal implications.

## Data Availability

The data supporting the findings of this case report are available from the corresponding author upon reasonable request.

## Research registration unique identifying number (UIN)

Not applicable. This is a case report.
